# Impact of metabolic syndrome on sex hormones and reproductive function: a meta-analysis of 2923 cases and 14062 controls

**DOI:** 10.18632/aging.202160

**Published:** 2020-12-01

**Authors:** Lihong Zhou, Liou Han, Mingyao Liu, Jixuan Lu, Shangha Pan

**Affiliations:** 1 Department of Endocrinology, The First Affiliated Hospital of Harbin Medical University, Harbin 150001, China; 2 Department of General Surgery, The First Affiliated Hospital of Harbin Medical University, Harbin 150001, China; 3 College of Life Science, Northeast Agricultural University, Harbin 150030, China; 4 Central Laboratory, The First Affiliated Hospital of Harbin Medical University, Harbin 150001, China

**Keywords:** metabolic syndrome, sex hormones, reproductive function, meta-analysis

## Abstract

Current evidence is inconsistent regarding the impact of metabolic syndrome (MetS) on sex hormones and reproductive function, and this meta-analysis aimed to illuminate the association. A literature search was conducted in public databases to identify all relevant studies, and study-specific standardized mean differences (SMD) and 95% confidence intervals (CI) were pooled using a random-effects model. Finally, 21 studies were identified with a total of 2923 MetS cases and 14062 controls. In males, MetS cases had a lower level of testosterone, inhibin B, total sperm count, sperm concentration, sperm normal morphology, sperm total motility, sperm progressive motility and sperm vitality, and a higher level of DNA fragmentation and mitochondrial membrane potential. In females, MetS cases had a higher level of testosterone. No significant difference was detected for follicle-stimulating hormone, luteinizing hormone, oestradiol, prolactin, anti-Müllerian hormone and semen volume in males, and for oestradiol, follicle-stimulating hormone, luteinizing hormone and progesterone in females. In conclusion, this meta-analysis indicated the impact of MetS on sex hormones and reproductive function, and MetS cases had a potential risk of infertility.

## INTRODUCTION

Metabolic syndrome (MetS) is composed of a constellation of metabolic disorders, including hypertension, dyslipidemia, abdominal obesity and insulin resistance or glucose intolerance [1, 2]. In spite of the association between MetS and health problems, its influence on human reproductivity has yet to be discussed [3]. First, as the typical characteristics of MetS, obesity, dyslipidemia and insulin resistance are thought to have an adverse impact on female reproductivity for impaired endometrial receptivity and compromised embryo development [4]. Second, MetS has a relatively high coincidence of the endocrine syndrome of polycystic ovary syndrome (PCOS). Infertility and cardio-metabolic disorders are more common in PCOS, which has an incidence of 5~15% in female [5]. However, the study by Mulder et al. demonstrated that infertile women had a higher level of cholesterol, low density lipoprotein cholesterol, triglycerides and body mass index, but blood pressure, fasting insulin, insulin resistance and fasting glucose showed no significant difference between fertile and infertile women [5]. Considering MetS as a set of these clinical conditions, the results further confused us regarding the association between MetS and female reproductivity.

On the other hand, as the constituent part of MetS, obesity could dysregulate the sex hormones, and cause oxidative stress damage of the semen microenvironment, sperms and interstitial glands [6, 7]. However, the MacDonald et al. study suggested no significant impact of BMI on the semen parameters, while the Sermondade et al. study found a higher incidence of azoospermia or oligozoospermia in the overweight and obese cohort [8, 9]. Moreover, diabetes, hypertension and dyslipidemia could down-regulate the secretion of testosterone, damage the testiculus and erectile function, and finally affect male fertility [10–12].

As a composite syndrome, MetS is deduced in association with the development of human reproductivity in view of its constituent disorders. Previous studies demonstrated a negative correlation between MetS and blood testosterone levels, while it was positively associated with oestrogen levels [13, 14]. However, the impact of MetS on reproductive function and other sex hormones is still controversial, and has not been clearly illuminated. Thus, this meta-analysis aimed to clarify the impact of MetS on reproductive function and sex hormones.

## RESULTS

### Characteristics of included studies

**Figure 1:**
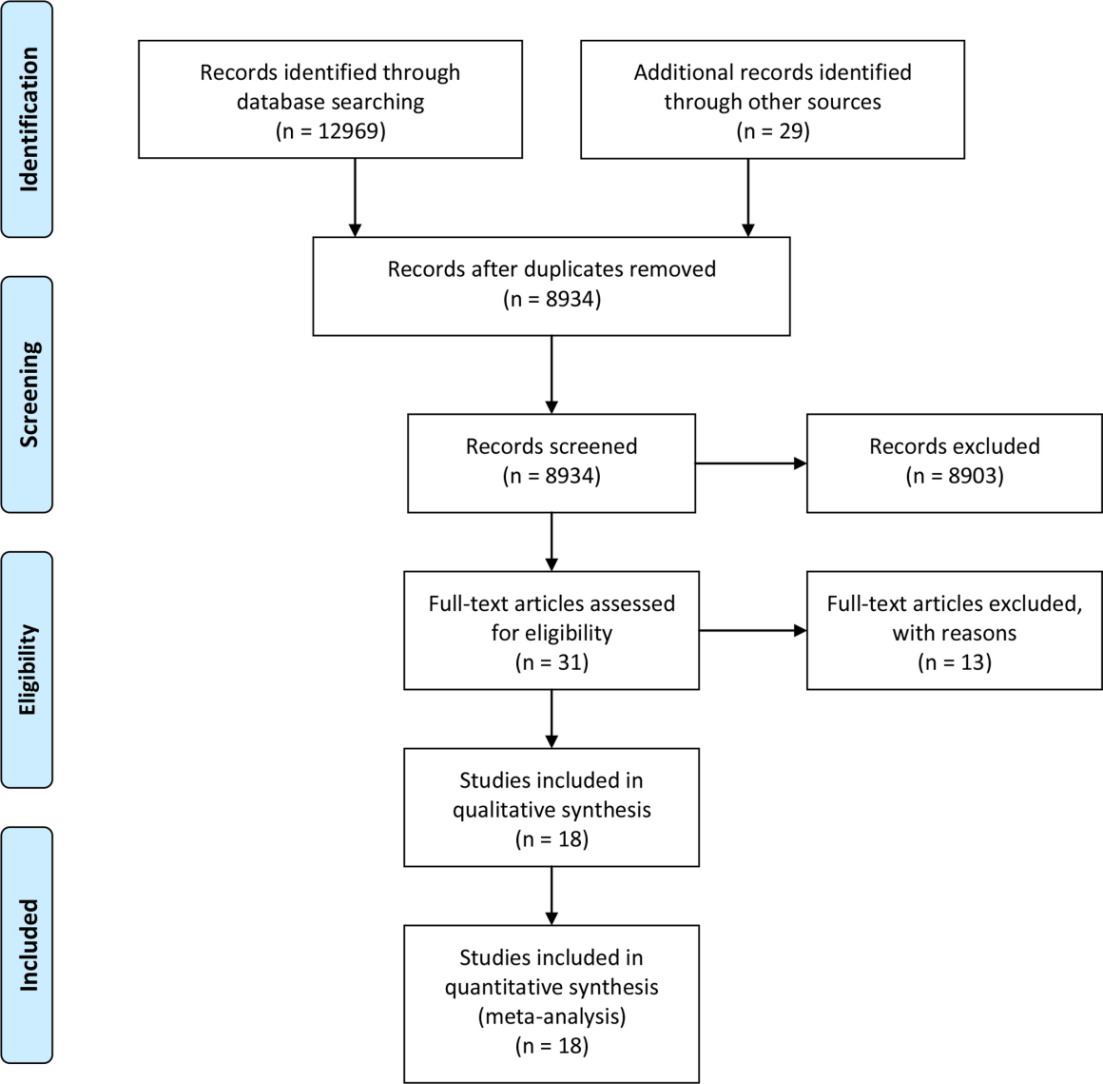
**Flowchart of literature search.**

The literature search retrieved 12998 records: 7822 from PubMed, 5147 from Embase, and 29 from other sources (Figure 1). After removing the duplication and unrelated records, 18 records (21 studies) were included into the meta-analysis [15–32] (Supplementary Table 1). Finally, we included a total of 2923 MetS cases and 14062 controls. The Ehala-Aleksejev et al. study investigated the male partners of fertile and infertile couples respectively, while the Natah et al. study and the Olszanecka et al. study focused on the premenopausal and postmenopausal women respectively. Thus, these studies were internally divided into two individual studies. Moreover, 13 studies focused on male cohort, 8 on female cohort, 5 on infertile cohort, 4 on postmenopausal female cohort, and 2 on PCOS cohort. None of the studies were prospective designed. Ten studies were conducted in Europe, 8 in Asia and 3 in Africa. In the assessment of methodological quality, the included studies reached a mean NOS score of 6.58.

### Impact of MetS on sex hormones in males

Comparatively, MetS cases had a lower level of testosterone (T) (n=8; SMD (95% CI): -5.00 (-8.48 to -1.52)) and inhibin B (InhB) (n=4; SMD (95% CI): -2.95 (-5.19 to -0.71)). No significant difference was found in follicle-stimulating hormone (FSH) (n=8; SMD (95% CI): -0.37 (-0.89 to 0.15)), luteinizing hormone (LH) (n=7; SMD (95% CI): -0.16 (-2.23 to 1.92)), oestradiol (E2) (n=5; SMD (95% CI): 0.62 (-2.09 to 3.33)), prolactin (PRL) (n=3; SMD (95% CI): 0.04 (-0.11 to 0.20)), and anti-Müllerian hormone (AMH) (n=2; SMD (95% CI): -0.92 (-2.06 to 0.22)) (Table 1). No obvious publication bias was detected.

**Table 1:** Meta-analyses of the impact of metabolic syndrome on peripheral sex hormones and reproductive function.

**Variables**	**No. of included studies**	**No. of cases**		**SMD (95% CI)**	***I^2^* (%)**	***P* in Egger's test**
		**MetS**	**Control**			
**Male**						
Follicle-stimulating hormone (FSH)	8	1064	4339	-0.37 (-0.89 to 0.15)	97	0.432
Testosterone (T)	8	1064	4339	-5.00 (-8.48 to -1.52)	100	0.102
Luteinizing hormone (LH)	7	1014	4309	-0.16 (-2.23 to 1.92)	100	0.192
Oestradiol (E2)	5	695	3848	0.62 (-2.09 to 3.33)	100	0.308
Prolactin (PRL)	3	175	1680	0.04 (-0.11 to 0.20)	0	0.760
Inhibin B (InhB)	4	245	1498	-2.95 (-5.19 to -0.71)	99	0.091
Anti-Müllerian hormone (AMH)	2	148	1356	-0.92 (-2.06 to 0.22)	95	-
Semen volume	11	891	4339	-0.51 (-2.18 to 1.16)	100	0.097
Total sperm count	6	729	3766	-0.66 (-1.26 to -0.05)	97	0.461
Sperm concentration	12	1776	11849	-0.85 (-1.55 to -0.16)	99	0.298
Sperm normal morphology	10	1717	11778	-0.56 (-0.93 to -0.19)	97	0.418
Sperm total motility	7	1536	10097	-0.68 (-1.30 to -0.05)	99	0.618
Sperm progressive motility	10	1276	9469	-0.54 (-0.91 to -0.17)	94	0.089
Sperm vitality	4	138	217	-0.78 (-1.00 to -0.55)	0	0.123
DNA fragmentation	4	128	197	0.69 (0.46 to 0.93)	0	0.304
Mitochondrial membrane potential (MMP)	2	54	55	0.89 (0.49 to 1.28)	0	-
**Female**						
Oestradiol (E2)	8	853	2071	0.04 (-0.19 to 0.28)	80	0.399
Follicle-stimulating hormone (FSH)	8	853	2071	-0.20 (-0.48 to 0.07)	86	0.300
Luteinizing hormone (LH)	5	631	1820	0.03 (-0.28 to 0.34)	82	0.482
Progesterone (P)	1	159	620	-0.13 (-0.30 to 0.05)	-	-
Thyroid-stimulating hormone (TSH)	2	558	1270	0.22 (-0.03 to 0.48)	76	-
Testosterone (T)	4	642	1799	0.14 (0.02 to 0.26)	23	0.412

MetS, metabolic syndrome; No., number; SMD, standardized mean difference; CI, confidence interval.

### Impact of MetS on semen quality in males

Comparatively, MetS cases had a lower level of sperm total count (n=6; SMD (95% CI): -0.66 (-1.26 to -0.05)), sperm concentration (n=12; SMD (95% CI): -0.85 (-1.55 to -0.16)), normal sperm morphology (n=10; SMD (95% CI): -0.56 (-0.93 to -0.19)), total sperm motility (n=7; SMD (95% CI): -0.68 (-1.30 to -0.05)), progressive sperm motility (n=10; SMD (95% CI): -0.54 (-0.91 to -0.17)), and sperm vitality (n=4; SMD (95% CI): -0.78 (-1.00 to -0.55)) (Table 1). MetS cases had a higher level of deoxyribonucleic acid (DNA) fragmentation (n=4; SMD (95% CI): 0.69 (0.46 to 0.93)), and mitochondrial membrane potential (MMP) (n=2; SMD (95% CI): 0.89 (0.49 to 1.28)). There found no obvious difference in semen volume (n=11; SMD (95% CI): -0.51 (-2.18 to 1.16)). No obvious publication bias was detected.

### Impact of MetS on sex hormones in females

Comparatively, MetS cases had a higher level of T (n=4; SMD (95% CI): 0.14 (0.02 to 0.26)) (Table 1). There found no obvious difference in E2 (n=8; SMD (95% CI) 0.04 (-0.19 to 0.28)), FSH (n=8; SMD (95% CI): -0.20 (-0.48 to 0.07)), LH (n=5; SMD (95% CI): 0.03 (-0.28 to 0.34)), progesterone (P) (n=1; SMD (95% CI): -0.13 (-0.30 to 0.05)), and thyroid-stimulating hormone (TSH) (n=2; SMD (95% CI): 0.22 (-0.03 to 0.48)). No obvious publication bias was detected.

## DISCUSSION

Infertility is characterized by the failure to conceive after more than one year’s unprotected intercourse [33]. Approximately, one in six couples suffers from infertility across the world, which also leads to a decrease in life quality [34]. Among the infertile couples, 50% was caused by the female-related factors, while 20% by male-related factors and 30% by unexplained factors [35].

Recently, the pathogenesis of female infertility is considered not limited to the endocrine or reproductive systems. Furthermore, female infertility have been found to coincide with several metabolic disorders [36]. Infertile females have a higher incidence of hyperlipemia, and are at high risk of pregnant hypertension after medically assisted reproduction [37]. The mechanism is still not illuminated. The Banuls et al. study found an increasing level of lipolysis in the follicular fluid of MetS patients, as well as an elevated level of lower high-density lipoprotein, triglyceride and cholesterol. This might lead to the alteration in embryo development [38]. Growing evidence suggests an association between MetS and female infertility. However, it is confused by the limitation in sample size and the inconsistency in measuring methods. In this meta-analysis, we only found a higher level of testosterone in MetS women. High testosterone level has been reported in association with female infertility [39]. Due to the lack of relevant studies, we failed to investigate the impact of MetS on female reproductive cells.

Male hypogonadism usually accompanies with testosterone deficiency, spermatogenesis impairment, and metabolic disorders like diabetes and obesity [40]. In recent years, it is classified into “organic hypogonadism” and “functional hypogonadism” [41]. Organic hypogonadism is characterized by an irreversible pathological impairment, and testosterone replacement therapy is considered low to the benefits. By far, functional hypogonadism is the most common form of hypogonadism in adulthood, characterized by no recognizable pathological alteration in the reproductive system. Metabolic disorders are common among the individuals with hypogonadotropic hypogonadism or late-onset hypogonadism, and thus obesity, diabetes and MetS are hypothesized in association with sexual dysfunction [40]. Both the reproductive parameters and metabolic profiles could benefit from the testosterone supplementation treatment for hypogonadism or insulin sensitizer administration for the concomitant metabolic disorders [41]. These indicate a potential association between hypogonadism and MetS, but previous studies reached inconsistent results, probably caused by the limitation in sample size. Thus, this meta-analysis aimed to investigate the impact of MetS on male reproduction. Finally, we found that MetS patients had a significant lower level of several semen parameters, circulating FSH, T and inhibin B, while they had a higher level of MMP and DNA fragmentation in sperms.

The mechanism is complicated, and might attribute to the superimposed effects of the component factors of MetS, especially overweight and diabetes. Several studies have reported the association between overweight and the abnormalities in sex hormone levels or semen quality [9]. The DNA fragmentation in sperms was more common in obese males than normal-weight males [42]. Furthermore, overweight and obese individuals had an increasing level of oxidative stress and inflammation, which disturbed the spermatogenesis [43]. On the other hand, diabetes also demonstrated an association with the abnormalities in sex hormone levels and semen quality [44]. Metformin treatment could significantly improve the sperm concentration, the percentage of motile cells, the percentage of normal cells, LH and testosterone levels in the men suffering from type 2 diabetes mellitus [45, 46]. Thus, as the syndrome of these metabolic disorders, MetS was deduced in association with the development of infertility.

Despite of the first meta-analysis in this field, the limitations should not be ignored. First, partial included studies failed to include a large study cohort. Second, none of the included studies were prospective designed. Third, there found significant heterogeneity among studies, which probably attributed to the study-specific heterogeneity caused by sampling errors. Nevertheless, these limitations could not prevent us from arousing people’s attention to reproductive health especially among those with MetS.

Conclusively, this study indicated the impact of MetS on sex hormones and reproductive function, and MetS cases had a potential risk of infertility.

## MATERIALS AND METHODS

### Literature search

Public databases of Embase and PubMed were used to retrieve related studies from inception to July 16^th^, 2020. The combined key words were used: (‘metabolic cardiovascular syndrome’ OR ‘dysmetabolic syndrome’ OR ‘metabolic X syndrome’ OR ‘reaven syndrome’ OR ‘insulin resistance syndrome’ OR ‘syndrome X’ OR ‘metabolic syndrome’) AND (’follicle-stimulating hormone‘ OR ’testosterone‘ OR ’luteinizing hormone‘ OR ’oestradiol‘ OR ’prolactin‘ OR ’inhibin B‘ OR ’anti-Müllerian hormone‘ OR ’progesterone‘ OR ’thyroid-stimulating hormone‘ OR ’semen‘ OR ’sperm‘ OR ‘azoospermia’ OR ‘oligozoospermia’ OR ’ovum‘ OR ’germ cell‘ OR ’follicle’). We only included the studies published in English. Moreover, the references of relative publications were also reviewed for potential studies. Our meta-analysis has been authorized by the ethics committee of The First Affiliated Hospital of Harbin Medical University.

### Study inclusion

Lihong Zhou and Liou Han selected the studies seperatively. Studies were included if fulfilling the following criteria: (i) contained both MetS individuals and the controls; (ii) focused on any indicators of sex hormones, semen or ovum parameters; (iii) the indicator levels were provided. We excluded animal studies, case reports, reviews and abstracts without full texts.

### Data extraction

Lihong Zhou and Liou Han designed a standardized collection form to extract the data, and the authors resolved the differences by discussion. The following data were extracted from each included study: publication year, author, study location, study population, MetS diagnosis criteria, sample size, and measurement parameters. If the studies originated from the same location, the study area and duration were reviewed to remove duplication. In quality assessment of the included studies, we adopted the Newcastle-Ottawa Scale (NOS, 0~9).

### Statistical analysis

Some studies presented the parameters as the median or average with standard error (SD), inter-quartile range (IQR), range or 95% confidence interval (95% CI). Uniformly, we converted the parameters to average ± SD according to the previous method [47]. In the meta-analysis of the impact of MetS on selected parameters, the inverse variance method and the random-effects model were adopted to pool the standardized mean differences (SMD) with 95% CIs. *Q* test and *I^2^* statistic were used to estimate the heterogeneity among studies, and the *I^2^* of more than 50% was considered significant heterogeneity [48]. Egger’s test was conducted to evaluate potential publication bias [49]. Most statistical analyses were realized with the software of Review Manager 5.4, only Egger’s test was conducted using the software of STATA version 11.0. A two-tailed *P* value of less than 0.05 was considered statistically significant.

## Supplementary Material

Supplementary Table 1
