# Supplementary material for: Impact of metabolic syndrome on sex hormones and reproductive function: a meta-analysis of 2923 cases and 14062 controls
Source: Aging (Albany NY). 2020 Dec 1;13(2):1962–71. doi: 10.18632/aging.202160 (PMC7880347; doi:10.18632/aging.202160)
Supplement: Supplementary Table 1 [file aging-13-202160-s001.docx]

**Supplementary Table 1. Characteristics of included studies in this meta-analysis.**

| **Study** | **Area** | **Study population** | **MetS diagnosis criteria** | **No.**  **MetS** | **No.**  **Controls** | **Parameters** |
| --- | --- | --- | --- | --- | --- | --- |
| **Male** |  |  |  |  |  |  |
| Lotti 2013 | Florence, Italy | Male members of infertile couples | International Diabetes Federation (IDF) criteria in 2009 | 27 | 324 | FSH, LH, PRL, TSH, T, semen volume, sperm concentration, sperm progressive motility, sperm normal morphology |
| Leisegang 2014 | Western Cape, South Africa | MetS and controls (24~67y) | International Diabetes Federation (IDF) criteria in 2009 | 24 | 26 | Semen volume, sperm concentration, total sperm count, sperm progressive motility, sperm total motility, sperm vitality, MMP, DNA fragmentation |
| Elsamanoudy 2016 | Mansoura, Egypt | Fertile MetS and controls (average 39~40y) | International Diabetes Federation (IDF) criteria in 2009 | 38 | 45 | Semen volume, sperm concentration, sperm progressive motility, sperm vitality, sperm normal morphology, DNA fragmentation |
| Leisegang 2016 | Bellville and Stellenbosch, South Africa | MetS and controls (25~65y) | International Diabetes Federation (IDF) criteria in 2009 | 32 | 42 | Semen volume, sperm concentration, total sperm count, sperm vitality, sperm progressive motility, sperm total motility, MMP, DNA fragmentation |
| Pilatz 2016 | Giessen, Germany | MetS and controls (30~62y) | National Cholesterol Education Program (NCEP) criteria in 2001 and International Diabetes Federation (IDF) criteria in 2009 | 27 | 27 | Semen volume, sperm concentration, sperm progressive motility, sperm normal morphology |
| Ventimiglia 2016 | Milan, Italy | Primary infertile men (average 36y) | National Cholesterol Education in Program (NCEP) criteria in 2004 | 128 | 1209 | FSH, LH, InhB, AMH, T, E2, PRL, TSH, semen volume, sperm concentration, sperm progressive motility, sperm normal morphology, total sperm count |
| Ventimiglia 2017 | Milan, Italy | Secondary infertile men (22~68y) | National Cholesterol Education in Program (NCEP) criteria in 2004 | 20 | 147 | FSH, LH, InhB, AMH, T, E2, PRL, TSH, semen volume, sperm concentration, sperm progressive motility, sperm normal morphology |
| Ehala-Aleksejev 2018 (1) | Tartu, Estonia | Partners of pregnant women (FM) (average 32y) | National Cholesterol Education in Program (NCEP) criteria in 2004 | 29 | 209 | Semen volume, sperm concentration, total sperm count, sperm total motility, sperm normal morphology, FSH, LH, T, E2 |
| Ehala-Aleksejev 2018 (2) | Tartu, Estonia | Male partners of infertile couples (MPIC) (average 33y) | National Cholesterol Education in Program (NCEP) criteria in 2004 | 471 | 2171 | Semen volume, sperm concentration, total sperm count, sperm total motility, sperm normal morphology, FSH, LH, T, E2 |
| Siddiqui 2018 | Saudi Arabia | T2DM (30~80y) | International Diabetes Federation (IDF) 2006 | 292 | 137 | T, FSH, LH |
| Chen 2019 | Taipei, China | Participants of reproductive age (average 32~34y) | Harmonized criteria in 2009 | 885 | 7510 | Sperm concentration, sperm total motility, sperm progressive motility, sperm normal morphology |
| Elfassy 2020 | France | Male partners of infertile couples (18~45y) | Harmonized criteria in 2009 | 47 | 112 | T, InhB, FSH, LH, E2, semen volume, sperm concentration, total sperm count, sperm progressive motility, sperm total motility, sperm vitality, sperm normal morphology, DNA fragmentation |
| Saikia 2020 | Gauhati, India | MetS and age-matched controls (20~40y) | International Diabetes Federation (IDF) criteria in 2005 | 50 | 30 | FSH, T, InhB, total sperm count, semen volume, sperm total motility, sperm progressive motility, sperm normal morphology |
| **Female** |  |  |  |  |  |  |
| Stefanska 2012 | Bydgoszcz, Poland | Postmenopausal Caucasian women (46~60y) | International Diabetes Federation (IDF) criteria in 2006 | 148 | 172 | FSH, E2, TSH |
| Güdücü 2013 | Turkey | Postmenopausal women (47~74y) | National Cholesterol Education in Program (NCEP) criteria in 2001 | 22 | 61 | E2, FSH, T, LH |
| Natah 2014 (1) | Babylon, Iraq | Premenopausal women (35~65y) | National Cholesterol Education in Program (NCEP) criteria in 2001 | 20 | 22 | E2, FSH, LH |
| Natah 2014 (2) | Babylon, Iraq | Postmenopausal women (35~65y) | National Cholesterol Education in Program (NCEP) criteria in 2001 | 21 | 18 | E2, FSH, LH |
| Olszanecka 2016 (1) | Krakow, Poland | Hypertensive premenopausal women (40~60y) | International Diabetes Federation (IDF) criteria in 2009 | 41 | 48 | T, E2, FSH |
| Olszanecka 2016 (2) | Krakow, Poland | Hypertensive postmenopausal women (40~60y) | International Diabetes Federation (IDF) criteria in 2009 | 32 | 31 | T, E2, FSH |
| Chang 2019 | China | POCS women (20~40y) | ASRM-Sponsored PCOS consensus workshop group criteria in 2004 | 159 | 623 | LH, FSH, P, E2, T |
| He 2019 | China | PCOS women (average 28y) | International Diabetes Federation (IDF) criteria in 2009 | 410 | 1098 | FSH, LH, E2, T, TSH |

MetS, metabolic syndrome; PCOS, polycystic ovary syndrome; T2DM, type 2 diabetes mellitus; FSH, follicle-stimulating hormone; T, testosterone; InhB, inhibin B; LH, luteinizing hormone; AMH, anti-Müllerian hormone; TSH, thyroid-stimulating hormone; E2, oestradiol; PRL, prolactin; P, progesterone; MMP, mitochondrial membrane potential; IQR, inter-quartile range; SD, standard error; No., number.
